# Hyperuricemia remodels the serum proteome toward a higher inflammatory state

**DOI:** 10.1016/j.isci.2023.107909

**Published:** 2023-09-14

**Authors:** Georgiana Cabău, Orsolya Gaal, Medeea Badii, Valentin Nica, Andreea-Manuela Mirea, Ioana Hotea, HINT-consortium, Cristina Pamfil, Radu A. Popp, Mihai G. Netea, Simona Rednic, Tania O. Crișan, Leo A.B. Joosten

**Affiliations:** 1 Department of Medical Genetics, “Iuliu Haţieganu” University of Medicine and Pharmacy, Cluj-Napoca, Romania; 2 Department of Internal Medicine, Radboudumc, Nijmegen, the Netherlands; 3 Department of Rheumatology, “Iuliu Haţieganu” University of Medicine and Pharmacy, Cluj-Napoca, Romania; 4 Department of Immunology and Metabolism, Life and Medical Sciences Institute, University of Bonn, Bonn, Germany

**Keywords:** Disease, Health sciences, Proteomics

## Abstract

Gout is an autoinflammatory disease triggered by a complex innate immune response to MSU crystals and inflammatory triggers. While hyperuricemia is an obligatory risk factor for the development of gout, the majority of individuals with hyperuricemia never develop gout but have an increased risk of developing cardiometabolic disorders. Current management of gout aims at MSU crystal dissolution by lowering serum urate. We apply a targeted proteomic analysis, using Olink inflammation panel, to a large group of individuals with gout, asymptomatic hyperuricemia, and normouricemic controls, and we show a urate-driven inflammatory signature. We add *in vivo* evidence of persistent immune activation linked to urate exposure and describe immune pathways involved in the pathogenesis of gout. Our results support a pro-inflammatory effect of asymptomatic hyperuricemia and pave the way for new research into targetable mechanisms in gout and cardiometabolic complications of asymptomatic hyperuricemia.

## Highlights

• Asymptomatic hyperuricemia is marked by a strong systemic inflammatory signature
• FGF-21 dampens urate crystals and palmitate-induced inflammation *in vitro*
• Proteomes of flaring gout and chronic tophaceous gout reveal targetable biomarkers
• Serum urate reduction attenuates the inflammatory signature of patients with gout

## Graphical abstract

**Figure:**
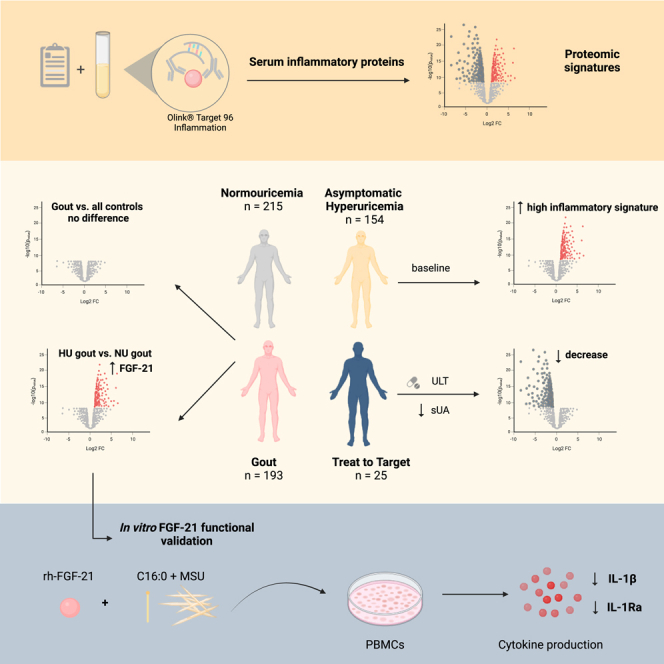

## Introduction

Gout is the most common inflammatory arthritis with a rising prevalence in the majority of developed nations.^1^ Hyperuricemia, defined as elevated serum urate concentrations, is the main risk factor for developing gout. Abnormally elevated urate precipitates into MSU (monosodium urate) crystals which deposit within joints and surrounding tissues triggering inflammation, manifested by painful acute arthritis known as the gout flare.^2^ The initial manifestation typically self-resolves within two weeks, followed by an asymptomatic period before a subsequent flare develops.^3^ Patients who have persistent hyperuricemia may experience chronic deposition of urate crystals in tophi and chronic joint inflammation, which can damage joints and impair articular motion.^4^ The greater mortality rate reported in patients with gout is attributed to the association of gout with other comorbidities such as cardiovascular disease, hypertension, metabolic syndrome, kidney stones, and chronic kidney disease.^5^

The pathogenesis of gout begins with hyperuricemia, and the likelihood of developing gout is correlated with serum urate concentrations in a dose-dependent manner. Hyperuricemia can be caused by factors that stimulate the overproduction of urate, including high-purine diets, myeloproliferative diseases, or other illnesses linked to a high rate of cellular turnover, as well as defects in excretion such as kidney dysfunction or certain diuretics. Cells of the innate immune system, primarily macrophages and monocytes, recognize MSU crystals as a damage-associated molecular pattern activating an inflammatory cascade resulting in the production of IL-1β through activation of the NLRP3 inflammasome, an intracellular danger signal sensor.^2^ Current treatment strategies in gout follow a treat-to-target approach using urate-lowering therapy aimed at the dissolution of MSU crystals, aiming for serum urate concentrations below 6 mg/dL as a treatment target.^6^

While this approach has supporting evidence in gout clinical trials, the treatment of asymptomatic hyperuricemia remains under debate.^7^^,^^8^^,^^9^ On the one hand, hyperuricemia is a necessary, but insufficient risk factor for the onset of gout. In the United States of America, around 20% of the population presents with hyperuricemia, but the majority never develop gout.^10^ Moreover, a significant percentage of individuals with hyperuricemia have asymptomatic crystal deposition.^11^^,^^12^ On the other hand, however, in the absence of gout flares, asymptomatic hyperuricemia is linked to cardiovascular disease, coronary heart disease, heart failure, stroke, an increased risk of cardiovascular death, increased overall mortality, cancer, and accelerated aging.^13^^,^^14^^,^^15^^,^^16^^,^^17^^,^^18^^,^^19^^,^^20^^,^^21^^,^^22^ According to some prospective studies, hyperuricemia leads to refractory hypertension, chronic kidney disease, metabolic syndrome, and type 2 diabetes.^21^^,^^23^^,^^24^^,^^25^^,^^26^^,^^27^ Experimental research demonstrates that soluble urate exerts direct pro-inflammatory effects on human PBMCs (peripheral blood mononuclear cells) and PBMCs from patients with gout and hyperuricemia produce higher levels of pro-inflammatory cytokines.^28^^,^^29^ Soluble urate primes cells for higher induction of cytokines via epigenetic reprogramming and aside from transcriptional upregulation of pro-inflammatory genes, urate specifically downregulates the transcription of *IL1RA*, enhancing inflammation, as IL-1Ra serves as the main IL-1β antagonist.^29^ This results in a pro-inflammatory phenotype of soluble urate-exposed monocytes and macrophages, which can lead to persistent immune activation and chronic inflammation.

In the present study, we aimed to: 1) characterize and compare the inflammatory proteomic signatures of gout and asymptomatic hyperuricemia, 2) functionally characterize the identified fibroblast growth factor 21 (FGF-21) biomarker in relation to cytokine production, and 3) monitor alterations in the proteomic profile of patients with gout following urate-lowering therapy. Our study adds to the expanding body of evidence supporting the inflammatory role of urate and provides new insights into the pathophysiological mechanisms of gout and their potential use as therapeutic targets.

## Results

### Baseline clinical and laboratory characteristics of the study groups

**Figure 1:**
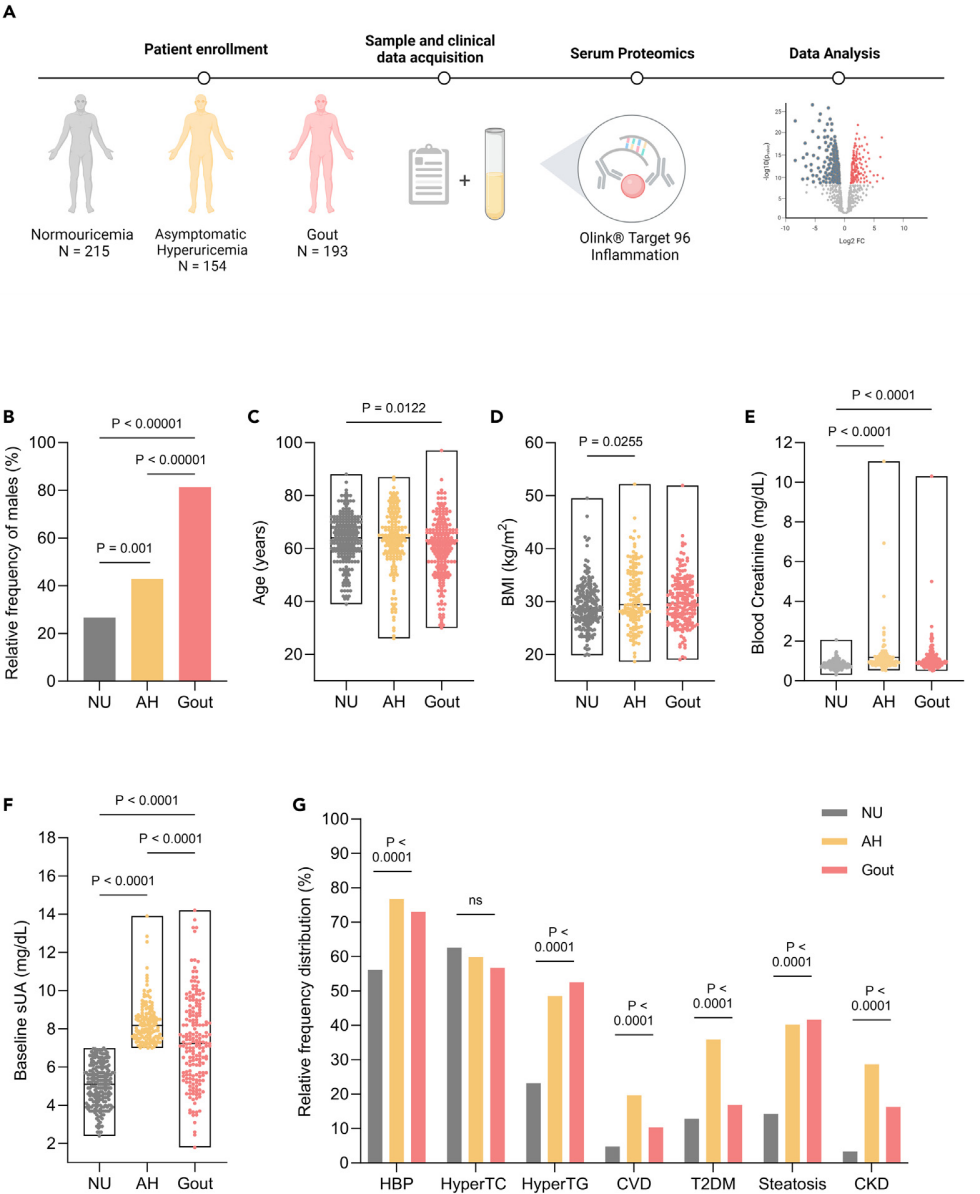
Baseline clinical and laboratory characteristics of the study groups (A) Study design. The image was created with BioRender.com. (B–F) Differences in (B) sex distribution, (C) age, (D) BMI, (E) blood creatinine, (F) serum uric acid, (G) comorbidities distribution among nomoruricemic, hyperuricemic individuals, and patients with gout. Floating bars (C–F) represent the range of values with line at median. (G) Bars (B, G) represent percentages. Kruskal-Wallis test was performed for (C–F) with Dunn’s test for multiple comparisons and the chi-squared test was used to test for differences in observed frequencies (B, G). Significance is reported as two-tailed p values (See also Table S1). Abbreviations: NU normouricemia; AH asymptomatic hyperuricemia, HBP high blood pressure, HyperTC hypercholesterolemia, HyperTG hypertriglyceridemia, CVD cardiovascular disease, T2DM type 2 diabetes mellitus, CKD chronic kidney disease.

We sought to characterize the inflammatory signatures of gout and asymptomatic hyperuricemia (AH) using patients and control sera. We included 193 patients with gout, 154 individuals with asymptomatic hyperuricemia, and 215 normouricemic controls (NU). Biological sample collection and baseline laboratory analysis were conducted at inclusion in the study (Figure 1A) (cf. STAR Methods). Differences between groups were found for age and sex. Male sex representation was higher in gout, 81.3% compared to 42.8% in AH and 26.6% in the NU group (Figure 1B). Patients with gout tended to be slightly younger (median 62 years, IQR 53.0–67.5) than NU controls (median 64 years, IQR 58.0–70.0), with no difference compared to AH (median 64 years, IQR 57.0–71.0) (Figure 1C). AH individuals had slightly higher BMI (median 29.4, IQR 27.1–35.1) compared to NU controls (median 28.5, IQR 26.4–31.7), with no difference compared to gout (median 29.6, IQR 26.8–33.1) (Figure 1D). Blood creatinine was significantly higher in gout (median 0.93 IQR 0.8–1.1) and AH (median 0.98, IQR 0.8–1.2) compared to NU controls (median 0.8, IQR 0.7–0.9) (Figure 1E). Baseline serum urate concentrations ranged from 2.4 to 6.9 with a median of 5.1 mg/dL in the NU controls groups, 7.0 to 13.9 with a median of 8.1 mg/dL for hyperuricemic individuals, and 1.8 to 14.2 with a median of 7.2 mg/dL for patients with gout (Figure 1F). There were significant differences in the prevalence of comorbidities, with AH and gout having higher occurrences of high blood pressure, hypertriglyceridemia, cardiovascular disease, type 2 diabetes mellitus, steatosis, and chronic kidney disease (Figure 1G; Table S1).

### Serum proteomic signatures of gout and hyperuricemia

**Figure 2:**
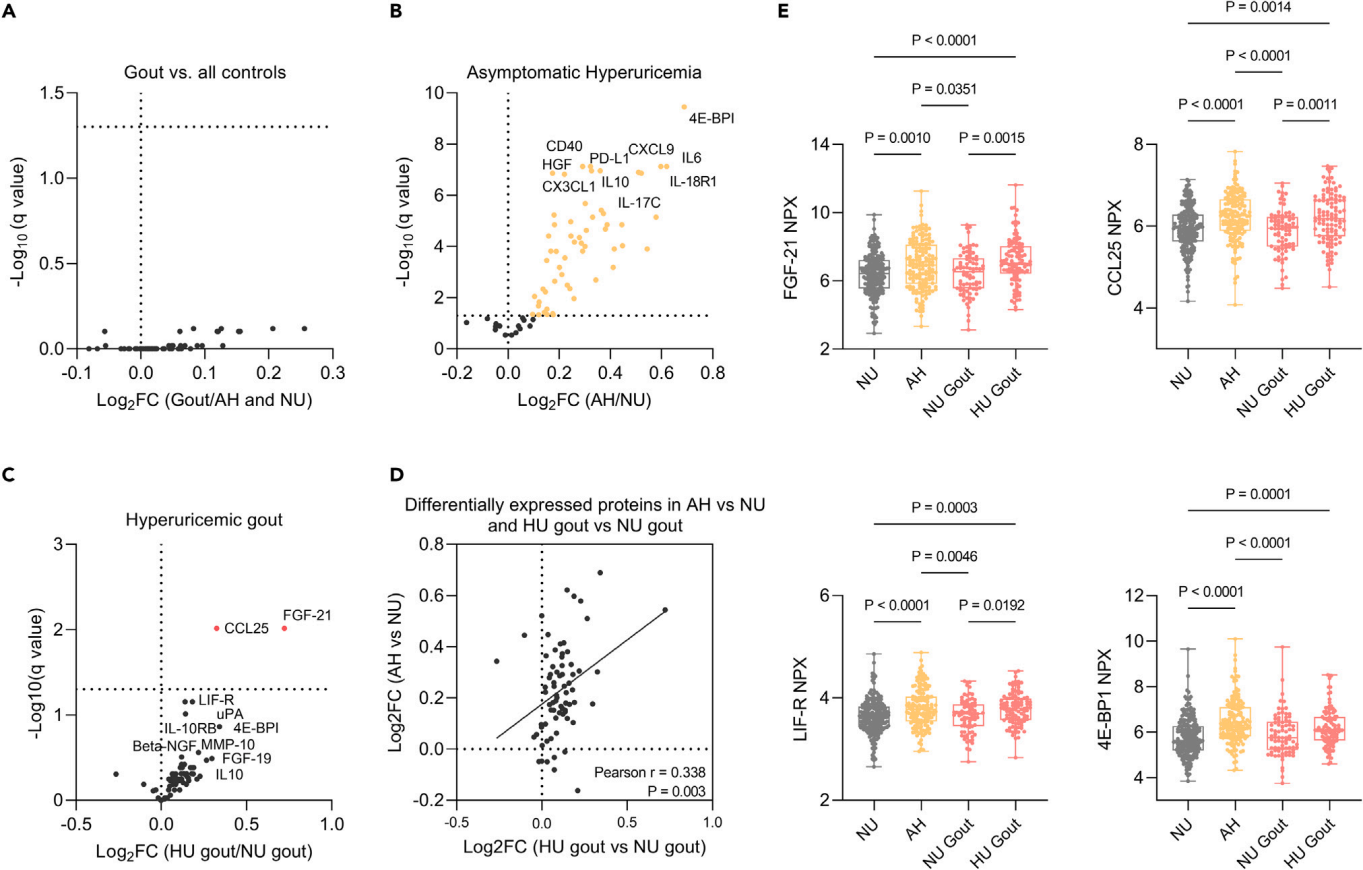
Serum proteomic signatures of gout and asymptomatic hyperuricemia (A) No difference in protein expression between gout versus all controls (NU and AH individuals). (B) Volcano plot showing proteins upregulated in AH compared to NU controls. (C) Proteins upregulated in hyperuricemic patients with gout compared to normouricemic patients with gout. (D) Top 10 proteins are labeled (D) Correlation of differences between AH vs. NU and HU gout vs. NU gout. (E) Targeted analysis of the two proteins upregulated in HU gout compared to NU gout, FGF-21, CCL25 and two nominally significant proteins, LIF-R and 4E-BP1. Volcano plots for (A–C) are shown. Associations with q values <0.05 were considered significant, Welch multiple t test, FDR 5%. Boxplots (E) show line at median and the 75^th^ and 25^th^ percentiles, whiskers show the range of values. Welch’s ANOVA with Games-Howell’s multiple comparisons test was used to test for differences between groups (See also Figure S1; Table S2). Abbreviations: NU normouricemia; AH asymptomatic hyperuricemia.

To determine the serum inflammatory proteomic signature of our study groups, targeted proteomic analysis was performed using the Olink Target 96 inflammation panel. We first investigated the proteomic profile difference between patients with gout and all controls (comprising both AH and NU individuals). Across the 73 proteins, we found no differentially expressed biomarkers between the two groups (Figure 2A). However, when we investigated the serum proteome profiles of AH versus NU individuals, we identified a marked inflammatory signature in the AH group, consisting of 58 significantly differentially expressed proteins (Figure 2B; Table S2).

The most upregulated protein in the AH group was the mTOR effector 4E-BP1, followed by IL-18R1. The concentrations of IL-6 and other members of the IL-6 family of cytokines such as LIF-R (leukemia inhibitory factor receptor) and OSM (oncostatin M) were also upregulated in the AH group. Other upregulated proteins were the immunomodulatory IL-10 cytokine and its receptor subunit IL-10RB, which together act as a negative regulator to dampen the inflammatory response^30^; the TNF cytokine family and its receptors: the pleiotropic TNF cytokine, TNFSF14, the receptors CD40, TNFRSF9, and OPG (osteoprotegerin); and to a lesser extent the pro-inflammatory lymphotoxin (formerly known as TNF-β).^31^ Among other cytokines upregulated in AH, we found interferon-gamma (IFN-γ), IL-17A and IL-17C, IL-12B, and the receptor subunit IL-15RA. The proteomic signature of hyperuricemia was also comprised of a wide array of chemokines both inflammatory and homeostatic, as well as dual chemokines. They were represented by the inflammatory CC chemokines, monocyte chemoattractant proteins MCP-1, MCP-2, MCP-3, and MCP-4, the homeostatic CCL19, CCL25, and CCL28, and the dual chemokines CCL11 and CCL20, as well as the platelet chemokine CCL23.^32^ The upregulated CXC chemokines comprised the neutrophil chemoattractants CXCL1, CXCL6, CXCL8, and the IFN-γ-inducible CXCL9, CXCL10, and CXCL11 axis, the latter involved in immune cell differentiation, migration, and activation.^33^ The novel CX3CL1, fractalkine, described to have anti-apoptotic effects on immune cells and other cell types^34^ was induced in the AH group. Another group of inflammatory proteins we screened for were growth factors, among which hepatocyte growth factor (HGF) was one of the top 10 most upregulated proteins, followed by CSF-1, TGF-α, β-NGF, vascular endothelial growth factor A (VEGFA), FGF-21, LAP TGF-beta, NT-3, and FGF-19. Other immunoregulatory proteins overexpressed in AH were programmed death-ligand 1, CD8 antigen, S100A12, CDCP1, the T cell marker CD5, SLAMF1, STAMBP, SIRT2, CASP8, CST5, uPA (urokinase), and the proteinases MMP-1 and MMP-10.

Next, we wanted to test if the proteomic profile of AH is recapitulated in gout. We stratified gout samples based on serum urate (sUA) concentrations equal to 7 mg/dL or higher, here called hyperuricemic gout (HU gout) n = 104 and below 7 mg/dL, here called normouricemic gout (NU gout) n = 81. We found two proteins FGF-21 and CCL25 significantly overexpressed in HU patients with gout compared to NU gout and another 8 nominally significant (Figure 2C). We further tested whether the lack of significance was due to the smaller number of patients included in the analysis and we looked for the directionality of differences, assessed as log2-fold-changes, resulting from the comparison of the AH group versus NU and HU gout versus NU gout group (Figure 2D). We observed a convergent directionality with most protein differences correlating between the two groups (Pearson r = 0.338, p = 0.003). This was also recapitulated when we looked at FGF-21 and CCL25 which were significantly increased, while LIF-R and 4E-BP1 were nominally significant in HU gout: we observed significantly higher concentrations of these proteins in HU gout compared to NU or NU gout, but no difference was observed in HU gout compared to AH, nor in NU gout compared to normouricemia (Figure 2E). The lack of differences between gout and the total control group could be thus explained by the high concentrations of inflammatory markers associated with hyperuricemia, masking the effect of gout when we compared gout against all controls. This is also supported by analyzing the gout group against normouricemic controls, which revealed 39 differentially expressed inflammatory markers, of which 36 overlapped with the markers associated to AH (Figures S1A and S1B). Comparison of AH to gout revealed 14 markers that had higher levels in AH samples than in gout (Figure S1C).

### The hepatokine FGF-21 modulates gout-related inflammation

The most significantly upregulated protein in the hyperuricemic gout samples compared to normouricemic gout was the hepatokine FGF-21, which also correlated with sUA concentrations (r = 0.29, p < 0.0001) (Figures 2C and S1D). Previous studies showed FGF-21 to be a pleiotropic stress-inducible hormone with tissue and organ-specific immunometabolic benefits.^35^^,^^36^

We hypothesized that FGF-21 may be an adaptive regulator in hyperuricemia and gout-related inflammation. To assess a functional role, primary PBMCs from 21 volunteers were stimulated *ex vivo* with palmitate (C16) together with MSU, which mimic fatty acid accumulation that may result from metabolic changes, hypothesized to initiate the gout flare (cf. STAR Methods section).^2^

**Figure 3:**
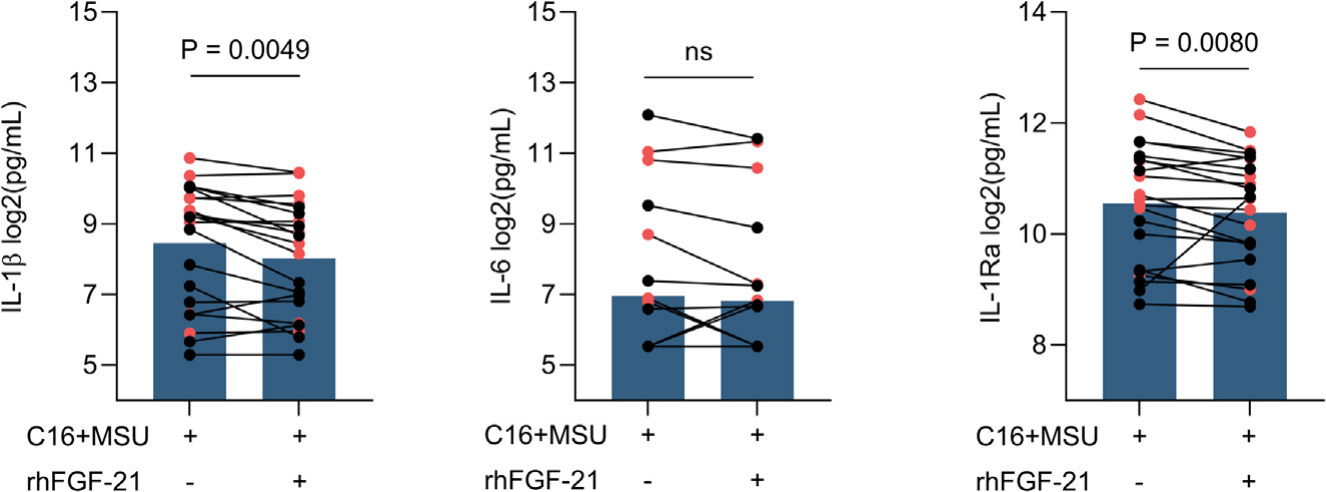
The hepatokine FGF-21 modulates gout-related inflammation Production of IL-1β, IL-1Ra, and IL-6 in response to stimulation with C16+MSU in the presence or absence of rhFGF-21 treatment. Data are representative for 21 individual samples, comprising of patients with gout (n = 10) labeled in red, and sex and age-matched controls (n = 11). Four independent experiments were performed. Dots and lines represent the paired samples under different conditions. Bars show means. Differences were tested using Wilcoxon matched pairs signed rank test. ns: not significant.

We found that in C16 and MSU-co-stimulated cells, treatment with rhFGF-21 decreased the production of IL-1β (p = 0.0049) and IL-1Ra (p = 0.0080), while it had no significant effect on IL-6 production (p = 0.2402) (Figure 3).

### Association of serum protein levels with gout flares and tophaceous gout

**Figure 4:**
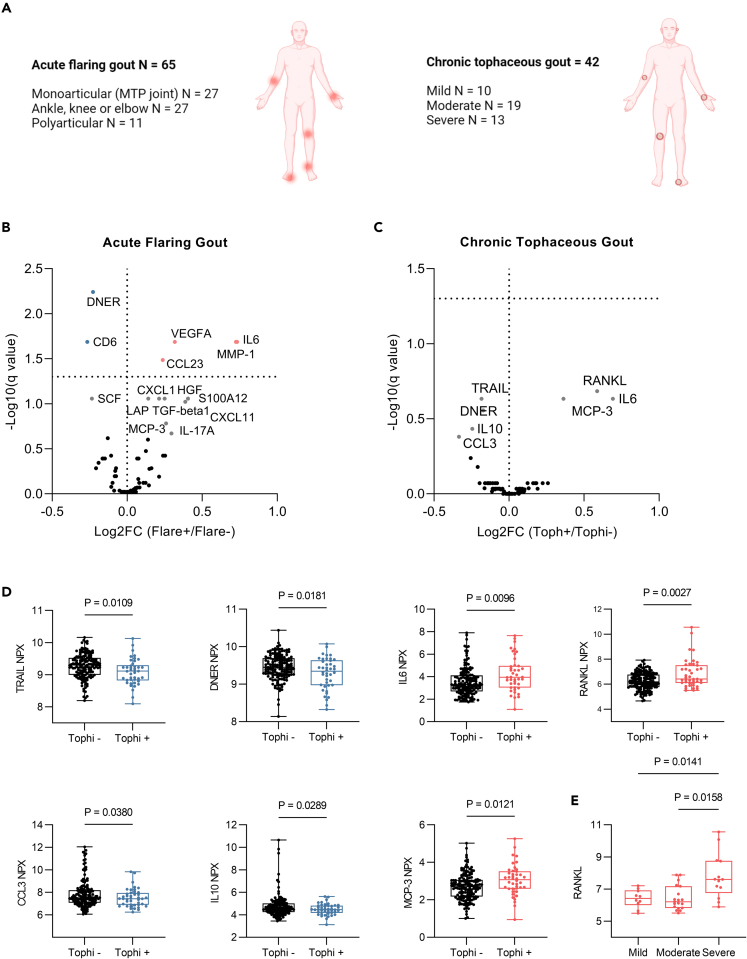
Association of serum protein levels with disease status in gout (A) Number of patients included in the analysis and their disease characteristics. The image was created with BioRender.com. (B and C) Volcano plots show differentially expressed proteins in acute flaring patients with gout compared to non-flaring patients (B) and chronic tophaceous gout compared to patients without tophi (C). Marked in red represent significantly increased proteins, marked in blue represent significantly decreased proteins, labeled, and marked in gray represent nominally significant proteins. Associations with q values <0.05 were considered significant, Welch multiple t test, FDR 5%. (D) Targeted analysis of the nominally significant proteins in tophaceous gout. (E) RANKL NPX levels in mild, moderate, and severe tophaceous gout. Boxplots show line at median and the 75^th^ and 25^th^ percentiles, whiskers show the range of values. Welch’s t test was used for two groups comparisons (D) and Welch’s ANOVA with Dunnett T3 for multiple testing correction (E) (See also Figures S2 and S3).

To better understand the mechanisms and molecular triggers of gout flares, we looked at whether there were any differences in the serum proteome of individuals who were experiencing flares. Out of the 193 patients with gout, 65 individuals presented and had serum collected during a flare. Out of the 65 patients, 27 presented with monoarticular disease, affecting the metatarsophalangeal joint, while another 27 had larger joints involved, such as the ankle, knee, or elbow, and 11 patients exhibited polyarticular disease (Figure 4A). Six significantly distinct proteins were identified in the serum proteomic profile of gout flares, these patients having elevated circulating concentrations of MMP-1, IL-6, VEGFA, and CCL23 and decreased DNER (Delta and Notch-like epidermal growth factor-related receptor) and CD6 levels (Figures 4B and S2A). Among the 10 proteins which were nominally significant before multiple testing correction, 8 proteins (LAP-TGF-beta, S100A12, HGF, CXCL1, CXCL11, MCP-3, IL-17A, and stem cell factor) showed significant differences in a targeted analysis between flaring patients and non-flaring patients, with the latter showing decreased levels (Figure S2B). We next looked at whether these markers correlated with the extent of joint involvement. IL-6, CCL23, VEGFA, MMP-1, S100A12, and DNER were strongly associated (p < 0.0001) (Table S3), and IL-6, CCL23, and S100A12 also had higher concentrations in polyarticular flares or when larger joints were affected compared to small joints (Figure S3).

The main source of joint injury, bone erosion, and pathological tissue remodeling in gout is thought to be the tophi, which arise as a result of repeated cycles of joint inflammation and resolution.^2^^,^^37^ We divided patients with gout into groups according to whether they had tophi (n = 42) or not (n = 151) to determine whether there was a proteomic signature associated with chronic tophaceous gout. Among the 42 patients diagnosed with chronic tophaceous gout, 10 exhibited mild disease, 19 had moderate disease, and 13 presented with severe chronic tophaceous gout (Figure 4A) (cf. STAR Methods). Nominally significant differences were observed for 7 proteins: TRAIL (TNF-related apoptosis-inducing ligand), DNER, IL-10, and CCL3 were reduced, and IL-6, RANKL (Receptor activator of nuclear factor kappa-Β ligand), and MCP-3 were elevated in samples of patients with tophaceous gout (Figures 4C and 4D). RANKL levels strongly correlated with disease severity (r = 0.37, p < 0.0001) (Table S4) and were higher in patients with severe tophaceous gout compared to mild and moderate disease (Figure 4E).

### Dynamic changes in the inflammatory signature of hyperuricemic gout, following urate-lowering therapy

**Figure 5:**
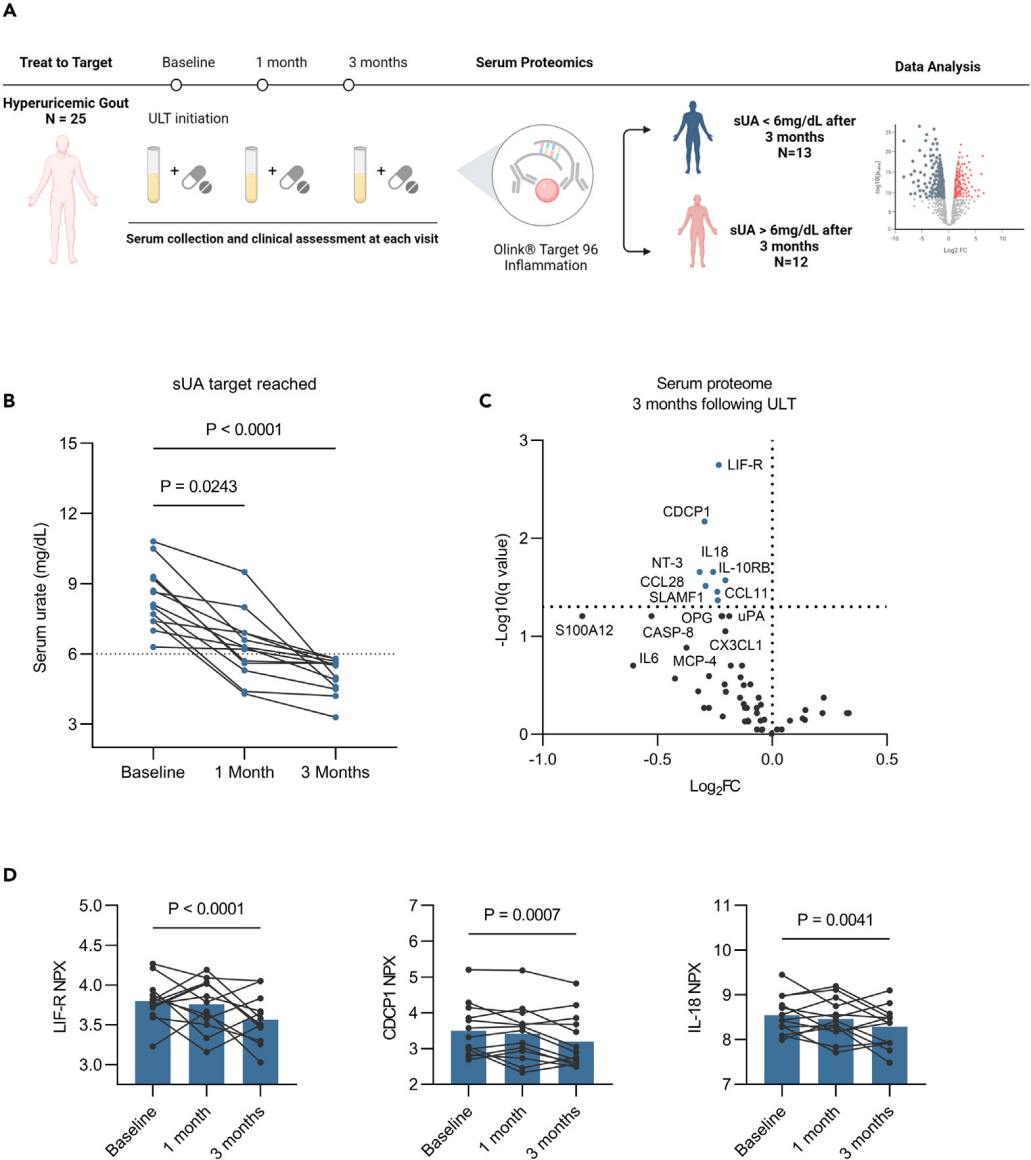
Dynamic changes in the inflammatory signature of hyperuricemic gout, following urate-lowering therapy (A) Study design. The image was created with BioRender.com. (B) Serum urate concentrations at baseline, 1 month and 3 months following ULT in patients that reached sUA target at 3 months. Dots and lines represent the paired samples at different time points. (C) Volcano plot showing significantly decreased proteins after 3 months of ULT compared to baseline levels, Welch’s multiple t test, FDR 5%. Labeled proteins under the significance threshold represent proteins that have a nominally significant decrease. (D) Three examples of the downregulated proteins after 3 months of ULT compared to baseline. Dots and lines represent the same sample at different time points. Bars show means (See also Table S5).

Lastly, we assessed whether the reduction of sUA concentrations can reverse the inflammatory profile observed in hyperuricemia. We included a separate group of 25 hyperuricemic patients with gout and collected serum and clinical chemistry data before, at one month, and at 3 months following initiation of allopurinol therapy (Figure 5A).

Out of 25 patients included, only 13 patients reached the sUA target of 6 mg/dL after three months, while the rest did not reach the target and were excluded from further analysis (Figures 5B and S4A). In the patients who achieved the sUA target, we compared the baseline urate concentration before urate-lowering therapy (ULT) and three months later for the proteins that were differentially expressed in asymptomatic hyperuricemia. Eight proteins out of 58 had significantly lower levels after three months of ULT, with LIF-R (q = 0.0017), CDCP1, IL-18, NT-3, IL-10RB, CCL28, CCL11, and SLAMF1 being the most highly downregulated (q = 0.0017) (Figures 5C and 5D; Table S5). No difference was observed after only one month of urate-lowering therapy (Figure S4B).

## Discussion

In this study, we performed targeted proteomics analysis to identify inflammatory proteins in large cohorts of patients with gout, individuals with asymptomatic hyperuricemia, and normouricemic individuals. We show a marked inflammatory signature in samples of individuals with AH compared to NU controls. Of high interest, no differentially expressed proteins were observed in samples of patients with gout versus all controls, which included asymptomatic hyperuricemia and normouricemic individuals. This could be explained by a urate-dependent effect which drives inflammation both in asymptomatic hyperuricemia as well as in gout. Indeed, we can observe a very similar inflammatory protein signature when stratifying patients with gout based on the presence of hyperuricemia. Although the mechanisms of immune activation and inflammation in gout are more established, asymptomatic hyperuricemia is less understood and still lacks an agreement concerning its pro-inflammatory effects and subsequent management. Here, we show that the proteomic signature of asymptomatic hyperuricemia recapitulates previous findings in gout and in studies of soluble urate-driven inflammation, as will be discussed in the following section. This report adds to the growing body of knowledge which suggests that AH may be pathogenetically linked to persistent immune activation, chronic inflammation, and to the subsequent increased prevalence of cardiometabolic comorbidities seen in hyperuricemic individuals.

The most upregulated protein identified in AH compared to NU, 4E-BP1, is a well-known mTOR substrate with a crucial role in mTORC1 signaling, which was shown to reprogram macrophages toward a more pro-inflammatory phenotype by translational repression of anti-inflammatory proteins.^38^ In line with this, soluble urate priming of human monocytes was shown to activate mTOR leading to a higher inflammatory response.^39^ The IL-1 signaling member IL-18 and its receptor IL-18R1 and the IL-6 family of cytokines members IL-6, LIF-R, and OSM were also elevated in AH, which is in line with major studies implicating IL-1-driven inflammation and IL-6 signaling in the pathogenesis of gout.^40^ IL-18 signals by binding to its receptor IL-18R1 and has been shown to be secreted in an inflammasome-independent way, by caspase-8 cleavage,^41^ with caspase-8 also being upregulated in AH sera in this study. IL-18 is reported to be elevated in patients with gout, associated with serum urate levels and postulated to contribute to the development of cardiovascular comorbidities.^42^ The TNF superfamily showed a strong upregulation in the AH group. In line with this, TNF production was shown to be induced by urate in rat vascular smooth muscle cells, an effect that was blocked by the addition of antioxidants, arguing for a pro-atherogenic effect of urate.^43^ TNF and TNFSF14 have also been shown to be induced by MSU crystals.^44^^,^^45^ A Th17 signature is also present in the urate inflammatory landscape identified in our study, characterized by elevated concentrations of the IL-17A, IL-17C, and IL-12B cytokine and the associated chemokines CXCL1, CXCL2, and CXCL6. More recently, Th17 responses have been described downstream of innate immune receptors signaling^46^ and NLRP3 activation, with the production of IL-1 cytokines potentiating Th17 responses and bridging innate and adaptive immunity.^47^ Previous studies have shown that patients with gout have increased levels of circulating Th17 cells,^48^ and that urate crystals in combination with an NF-κB priming signal induce Th17 polarization *in vitro.*^49^ Moreover, in MSU-induced joint inflammation in mice, blocking IL-17 attenuated the recruitment and activation of immune cells, as well as joint inflammation.^50^

Another marker that was elevated in hyperuricemic individuals was FGF21. Due to the observation that this was also the most upregulated protein in hyperuricemic patients with gout compared to NU gout, we further assessed the functional consequences of rhFGF-21 addition *in vitro* stimulation experiments using primary human PBMCS. Our study indicates that rhFGF-21 dampened cytokine production (Figure 3) and we hypothesize that FGF-21 may play a role in limiting gouty inflammation by decreasing the inflammatory response of the gout-specific stimulus, C16.0 and MSU. FGF-21 beneficial effects on carbohydrate and lipid metabolism have been shown to be mediated by the fatty acid sensor peroxisome proliferator-activated receptor γ (PPARγ).^51^ PPARγ is also expressed in immune cells and mediates its effect by a complex, ligand-dependent transrepression of inflammatory genes.^52^ Exposure to MSU crystals was shown to induce PPARγ in monocytes with authors arguing its implication in the resolution of flares.^53^ Elevated PPARγ expression was also demonstrated in synovial cells of acute patients with gout and gene variation in PPARγ coactivator 1β was associated with IL-1β production in patients with gout.^54^ Even though FGF-21 analogs are currently undergoing clinical trials and that FGF-21 has emerged as a viable therapeutic target for metabolic disorders, particular consideration should be given to reported side effects in relation to a potential use in gouty arthritis.^35^ Skeletal system side effects are frequently documented, with FGF-21 shown to mediate the detrimental effects leading to bone loss by altering the RANKL/OPG ratio.^55^^,^^56^^,^^57^ While the mechanisms of bone erosion in gout are incompletely understood, most studies point toward the implication of the RANKL/OPG pathway dysregulation, which may present a limitation to the potential use of FGF-21 in gouty arthritis.^58^^,^^59^^,^^60^

These observations are consistent with our findings on tophaceous gout (Figures 4C–4E) where we found that patients with tophi have higher circulating concentrations of the pro-osteoclastogenic RANKL and those with severe disease have even higher levels compared to mild or moderate disease, suggesting that RANKL plays a key role in bone erosion in gout. Furthermore, our research revealed that patients with tophaceous gout have lower concentrations of circulating TRAIL. It has been known that TRAIL can induce osteoclast differentiation via a TRAF-6-dependent mechanism, but there is increasing evidence for a paradoxical role of TRAIL in inhibiting RANKL-induced osteoclastogenesis by inhibition of lipid raft assembly required for TRAF-6 signaling.^61^^,^^62^ Osteoimmunological reports support a suppressive role of TRAIL in RANKL and M-CSF-induced osteoclast differentiation,^63^^,^^64^ with others showing the capacity of recombinant TRAIL to inhibit osteoclastogenesis and bone resorption via inhibition of RANKL signaling in a mouse model of collagen-induced arthritis.^62^ Other reports showed that recombinant TRAIL increased bone mass in an *in vivo* mouse model.^65^ It is noteworthy that dose-escalation urate-lowering therapy using allopurinol has been shown to prevent the progression of bone erosion in gout compared to conventional dosing; however, the reduction of bone loss was observed only after two years of treatment, with bone erosion initially progressing under treatment.^6^ For reversal of bone erosion to be achieved, severe sUA lowering to undetectable levels was required.^8^^,^^66^ These results highlight the need for additional interventions, making RANKL and TRAIL attractive therapeutic targets that could be used in combination with ULT to prevent joint damage and possibly reverse bone erosion, as both RANKL inhibitors, as well as rhTRAIL and activating antibodies of its death receptors, DR4 and DR5, are already undergoing human clinical trials. TRAIL is mostly used as a therapeutic target in cancer due to its potent ability to selectively induce apoptosis of cancer cells^67^ and RANKL-neutralizing antibodies showed efficacy in preventing osteoporotic^68^ and cancer-related fractures.^69^

The synovium during flares is characterized by infiltration of mononuclear cells, neutrophils, and lymphocytes. Following the phagocytosis of MSU crystals, monocytes become activated and release pro-inflammatory cytokines including IL-6 and IL-1β.^70^ Our results show that flaring patients exhibited elevated concentrations of circulating proteins that reflected higher levels of inflammation, immune infiltration, tissue remodeling, and angiogenesis (Figures 4B and S2). VEGFA has been previously associated with serum urate concentrations in a human genome-wide association study^71^ as well as in a mouse model of gout.^72^ VEGFA is a pro-angiogenic factor produced by synovial monocytes and fibroblasts upon stimulation by IL-1β, TGF-β, and other mediators^73^ and which can also act as a chemoattractant for monocytes and neutrophils.^74^ VEGFA can induce MMP-1 and other MMPs which promote angiogenesis by degrading the extracellular matrix and allowing for the migration of new cells within the synovium and proliferation of new blood vessels.^75^ Another pro-angiogenic factor, HGF, was also enriched in the sera of flaring patients with gout. Previous studies showed its expression in the synovium of rheumatoid arthritis and osteoarthritis patients and that inhibition of HGF suppressed progression of arthritis and bone destruction in SKG mice.^76^ Another recent report in gout showed that the HGF/MET pathway blockade in a model of MSU-induced arthritis resolved articular inflammation, tissue damage, and joint pain by enhancing neutrophilic apoptosis and reducing neutrophil infiltration, as well as decreasing CXCL1.^77^ CXCL1, a neutrophil attractant and activator of proteases, is a chemokine that was also found to be enriched in flaring patients in the present study. The interstitial collagenase MMP-1 has also been shown to be produced by synovial fibroblasts in response to MSU crystals.^78^ Previously, TGF-β has been shown to have context-dependent pro-inflammatory and anti-inflammatory roles, mediating urate-induced inflammation,^79^ as well as limiting acute gouty inflammation when added exogenously in a synovium-like air pouch mouse model.^80^ In a previous report investigating TGF-β in urate-induced inflammatory priming, we were not able to observe major differences in TGF-β or VEGFA levels during gout flares compared to inter-critical patients with gout.^79^ Possibly due to the different number of samples, in the present study, we were able to observe significant differences for VEGFA and nominally significant increase in TGF-β-LAP. This is in line with other studies showing TGF-β increase over the course of gout flares and potential involvement in the resolution of inflammation.^81^ We also found higher levels of chemokine CCL23, a strong chemotactic molecule for resting T cells and monocytes;^82^^,^^83^ CXCL11, chemotactic for activated T cells;^84^ and the monocyte chemotactic protein 3, MCP-3. IL-17A was also more elevated in our analysis of flaring versus inter-critical gout. IL-17A has been previously reported as a marker of acute gout, with γδT cell-derived IL-17A correlating with disease activity and IL-1β levels.^83^ Finally, the S100A12 protein was shown to be released by MSU-stimulated neutrophils^85^ and was independently shown to induce mast cell recruitment and activation,^86^ which has been hypothesized to play a role in the inflammatory cascade of acute gout.^70^

Lastly, to assess whether serum urate reduction would have the potential to reverse the elevated levels of inflammatory biomarkers presented here, we investigated the effect of ULT on the inflammatory status of patients with gout. No change of the inflammatory proteome has been observed after 1 month of ULT, despite a slight reduction in serum urate levels already present at this time. After three months of ULT, in patients who reached the target urate level of 6 mg/dL, eight inflammatory markers were significantly decreased, and seven others showed a nominally significant reduction. This shows that, indeed, interventions aimed at reducing urate concentrations do, in the long-term, also reduce inflammatory profile of the patients. We show that both a reduction in sUA concentration and a longer duration of treatment play a role in reversing the inflammatory status of patients with gout. It is tempting to speculate that further longer term maintenance of urate concentrations within normouricemic limits would ultimately lead to further diminishment of systemic inflammation. Epigenetic mechanisms that could lead to the inflammatory reprogramming of myeloid cells have been implicated in gouty inflammation and in response to soluble urate.^87^^,^^88^ From other studies showing innate immune memory induced by sterile metabolic stimuli, such as ox-LDL particles, we know that it is possible for inflammatory phenotypes of reprogrammed cells to not disappear even despite normalization of the initial trigger as in the case of cholesterol normalization using statins.^89^ Therefore, the persistence of elevated concentrations of some inflammatory markers in hyperuricemic individuals even at the time of urate normalization could potentially be explained by other long-term epigenetic processes that maintain inflammation and that may take longer time to reverse.

Earlier reports that looked at urate-lowering therapy have addressed the effect of urate in mediating adverse cardiovascular and renal events in relation to high blood pressure and showed improvements attributed to sUA reduction in adolescents^90^^,^^91^ but not in adults,^92^ while contrasting findings were also found for chronic kidney disease.^93^ An explanation for this discrepancy lies in the duration of treatment. For adolescents in the trials, the amount required for the benefit of ULT to be reached was 1 month and 2 months, respectively. For the adults in whom no improvement in systolic blood pressure was observed, the duration of treatment was 2 months. This could reflect the underlying biology of older individuals compared to younger individuals, but, as described previously, it could also be linked to urate-inducing long-term epigenetic modifications that imprint a pro-inflammatory phenotype on myeloid cells.^87^ Another explanation for the anti-inflammatory effect of allopurinol could lie in the inhibition of xanthine oxidoreductase, its therapeutic target, and the subsequent blocking of xanthine oxidase-derived reactive species of oxygen.^94^ Our data showing a long-term inflammatory potential of hyperuricemia in the absence of gout add an important argument that high uric acid concentrations, rather than urate crystals alone, are an important risk factor for cardiometabolic complications, and need to be addressed for the prevention of these life-reducing complications.

In summary, here we provide evidence for inflammatory consequences of urate exposure *in vivo.* We describe a strong and broad inflammatory signature associated with asymptomatic hyperuricemia which correlated with differences observed in patients with gout and hyperuricemia compared to normouricemic gout. The proteins identified here were also associated to clinically relevant phenotypes in gout, such as presence and severity of flares or tophi. Importantly, the increased expression of these proteins is partially reversible upon urate-lowering therapy, which argues for the causal effect of urate in the significant enrichment observed for these proteins in hyperuricemic individuals. Our findings represent a foundation for future functional studies to examine mechanistic relationships of these inflammatory proteins in gout and hyperuricemia, to explore their potential to be used as clinically relevant disease biomarkers, and for the development of therapies targeting asymptomatic hyperuricemia as a strategy to prevent cardiometabolic diseases.

### Limitations of the study

Some potential caveats of our research include the cross-sectional design for some of our sub-studies, which limits the ability to establish a causal relationship between serum urate levels, inflammation, and the development of cardiometabolic complications in AH and gout. Longitudinal studies could improve our understanding of these associations. By using a targeted proteomic approach, we may not have captured the inflammatory landscape in its entirety. Untargeted proteomic methods might offer a more comprehensive signature. Although we identified potentially targetable biomarkers in our study, further functional validations are needed to establish their role in the progression and resolution of the disease. While our study examines the impact of urate-lowering therapy on the inflammatory profile of gout, where we observed a decrease in inflammatory proteins paralleling a decrease in serum urate, other treatment-specific, urate-independent, anti-inflammatory mechanisms cannot be excluded. Lastly, our study did not account for all potential variables such as medication, BMI, lifestyle factors, and comorbidities which might impact inflammation profiles, as this may lead to overcorrection of data since hyperuricemia might be an underlying driver.

## STAR★Methods

### Key resources table

**Table:** 

REAGENT or RESOURCE	SOURCE	IDENTIFIER
**Biological samples**		

Human serum from gout patients, asymptomatic hyperuricemia individuals and normouricemic controls	Rheumatology clinic, Cluj-Napoca, Romania	N/A
Primary human peripheral blood mononuclear cells from gout patients, asymptomatic hyperuricemia individuals and normouricemic controls	Rheumatology clinic, Cluj-Napoca, Romania	N/A

**Chemicals, peptides, and recombinant proteins**		

Palmitate (C16)	Sigma-Aldrich	N/A
Monosodium urate crystals (MSU)	In-house as detailed in the main text	N/A
Recombinant human FGF-21	R&D Systems	Cat# 2539-FG

**Critical commercial assays**		

Human IL-1 beta/IL-1F2 DuoSet ELISA	R&D Systems	RD-DY201
Human IL-6 DuoSet ELISA	R&D Systems	RD-DY206
Human IL-1ra/IL-1F3 DuoSet ELISA	R&D Systems	RD-DY280
Olink® Target 96 Inflammation	Olink	https://olink.com/products-services/target/inflammation/

**Deposited data**		

Olink 96 Target Inflammation proteomics dataset	This study	Mendeley Data https://doi.org/10.17632/rhn9vsgk45.1

**Software and algorithms**		

GraphPad Prism 9	Graphpad Software	http://www.graphpad.com
RStudio v.4.2.1	RStudio, PBC	https://www.rstudio.com
BioRender	BioRender	https://biorender.com/

**Other**		

RPMI-1640 media	Sigma-Aldrich	Cat# R7638
Gentamicin	Sigma-Aldrich	Cat# G1397
Ficoll-Paque PLUS	Sigma-Aldrich	Cat# GE17-1440-03
GlutaMAX™ Supplement	ThermoFisher	Cat# 35050061

### Resource availability

#### Lead contact

Further information and requests for resources and reagents should be directed to and will be fulfilled by the lead contact, Leo Joosten (leo.joosten@radboudumc.nl).

#### Materials availability

This study did not generate new unique reagents.

### Experimental model and study participant details

#### Human subjects

In the present study participants were enrolled and recruited between 2016 and 2020 and attending the Rheumatology, Internal Medicine, Diabetes or Geriatrics clinics of the County Emergency Hospital of Cluj-Napoca, Romania. Ethical approval was obtained from the University of Medicine and Pharmacy of Cluj-Napoca, reference no. 425/2016. Informed consent was obtained from all individuals included. Participants were part of a larger study HINT, “Hyperuricaemia induced INflammation: Targeting the central role of uric acid in rheumatologic and cardiovascular diseases”, P_37_762, MySMIS 103587, a multi-omics study aiming to assess whether soluble urate induces epigenetic and transcriptional reprogramming in cells of innate immune system, leading to a hyperinflammatory state. None of the participants were part of previous procedures. Due to the association of age and sex with the serum inflammatory status, the proteomic data was corrected for age and sex (See quantification and statistical analysis). After proteomic analysis exclusion criteria (See proteomic analysis and data processing, STAR Methods section) the present study included a cross-sectional cohort comprising 193 patients with gout, 154 individuals with asymptomatic hyperuricemia and 215 normouricemic controls with sample and clinical data collected at baseline. In this cohort participants had a mean age of 62 years, ranging from 26 to 97, 48.5% were male and 97% were White from Romania. The participants were subject to treatment based on their respective chronic conditions and were not subject to drug or testing for the purpose of the present study (Please see clinicopathologic assessment of cohorts in STAR Methods details).

The study included another independent group comprised of 25 “Treat-to-Target” patients with gout following a longitudinal study design with repeated samples, clinical and laboratory data collected at baseline, after 1 month and after 3 months of initiating urate-lowering therapy (ULT). Participants were subject to treatment using allopurinol in the standard dosage of 100 to 300 milligrams (mg) per day at first and up to 400 mg after dosage stabilization. This group included patients with a mean age of 63 years ranging from 28 to 74, 81% were male and 100% were White from Romania. Of note, the treat-to-target study was designed for a 12-month follow-up, but the sample collection was compromised due to the epidemiologic circumstances of the COVID-19 pandemic. For this reason, the data presented here only shows the evolution over three months.

#### Primary cell culture

For *in vitro* experiments, blood was collected from another group of 10 patients with gout and 11 sex and age-matched controls. These participants had a mean age of 60 years, ranging from 34 to 80, 52% percent were male and 100% were White from Romania. Ethical approval was obtained from the University of Medicine and Pharmacy of Cluj-Napoca, reference no. 425/2016. Peripheral blood mononuclear cells (PBMCs) were isolated from whole blood by density gradient centrifugation using Ficoll-Paque PLUS (Sigma-Aldrich). For *ex-vivo* stimulation with FGF-21, PBMCs f were seeded in 96-well round-bottom microplates (Greiner Bio-One) at a density of 5 x 10^6^ cells/mL in cell medium RPMI-1640 (Sigma-Aldrich) supplemented with 1 mM pyruvate, 50 μg/mL gentamicin (Sigma-Aldrich) and 2 mM GlutaMAX (ThermoFisher Scientific). The cells were stimulated with either 50uM C16:0 (palmitate) together with 300ug/mL MSU crystals with 2ug/mL rhFGF-21 (recombinant human FGF-21) (R&D Systems) or medium control. MSU crystals were prepared in-house by diluting crystalline uric acid in a solution of NaOH: 1.0 gram of crystalline uric acid was solubilized in 200 ml of sterile water containing 24 grams of NaOH. The pH was adjusted to 7.2 using HCl. The solution became pyrogen-free after heating for 6 hours at 120°C. The solution was left to cool at room 30 temperature and stored at 4°C. Crystals produced were 5–25 μm in length. Plates were incubated at 37°C and 5% CO_2_ for 24h. After 24h incubation, plates were centrifuged, supernatants were removed and stored at -20°C until cytokine measurement was performed.

### Method details

#### Clinicopathologic assessment of cohorts

For the diagnosis of gout, patients fulfilled the 2015 American College of Rheumatology/European League Against Rheumatism (ACR/EULAR) criteria for the classification of gout, having a score of 8 or higher, or had crystal-proven gout with identification of MSU crystals in synovial fluid using compensated polarized light microscopy. For asymptomatic hyperuricemia, we defined hyperuricemia as serum urate concentration (sUA) greater or equal to 7 mg/dL in the absence, and without a history, of gout. For normouricemia, we included individuals with sUA below 7 mg/dL.

We collected participant information (reported in Figure 1) regarding age, sex, BMI, clinical chemistry including serum uric acid, total cholesterol (TC), triglycerides (TG), blood creatinine and medical history regarding cardiometabolic and renal comorbidities. Type 2 diabetes was based on repeated measurements of blood fasting glucose concentrations of 126 mg/dL or higher. Liver steatosis was evaluated by abdominal ultrasonography. Hypercholesterolemia (HyperTC) was defined as serum total cholesterol of 200 mg/dL or higher. Total triglyceride concentrations above 150 mg/dL were used as cut-off for hypertriglyceridemia (HyperTG). Repeated measurements of systolic blood pressure above 150 mmHg were used for diagnosing high blood pressure. Cardiovascular diseases (CVD) included myocardial infarction, stroke, transient ischemic attack and peripheral artery disease. Chronic kidney disease (CKD) was defined as reduced glomerular filtration rate for more than 3 months (GFR < 60 mL/min/ 1.73 m^2^). Chronic tophaceous gout diagnosis was determined by evaluating the extent and characteristics of the tophi, chronic arthropathy, joint inflammation and deforming features observed during the clinical examination and as previously described in the 2012 ACR guidelines for management of gout. The following parameters were clinically assessed: number of tophi, tophus drainage, tophus infection, extent of tissue destruction, size and growth rate, and joint inflammation. Mild disease was defined as simple chronic tophaceous gouty arthropathy limited to 1 joint and stable disease characterized by stable tophus size and slow growth, absence of drainage, low risk of infection, lack of aggressive tissue destruction and lack of severe chronic joint inflammation. Moderate disease was defined as simple chronic tophaceous gouty arthropathy affecting 2-4 joints and stable disease characterized by multiple, but stable and simple tophi as described above. Severe disease was defined as chronic tophaceous gouty arthropathy affecting more than 4 joints or more than 1 unstable, complicated tophus that presented drainage, a high risk of infection, accelerated growth, extensive tissue destruction and severe chronic tophaceous joint inflammation.

#### Proteomic analysis and data processing

A total of 732 serum samples from normouricemic (NU), asymptomatic hyperuricemia individuals (AH) and patients with gout that were collected at enrolment or at every visit for the treat-to-target group and stored at −80°C, were thawed on ice mixed by pipetting and randomized before plating on 96-well PCR microplate. Serum proteomic measurement was performed using Olink proximity extension assay, technology that uses qPCR for the simultaneous quantification of a pre-designed panel of proteins within a sample.^95^ For this study, we used the Olink® Target 96 inflammation panel.^96^ Proteomic assay, data normalization and quality control were performed at Olink Proteomics, Uppsala, Sweden. Briefly, the technology uses specific oligonucleotides-labelled antibodies against the target protein that upon binding come in proximity generating a PCR reporter sequence that allows quantification of protein abundance by Real-Time PCR (qPCR). Using sample and plate controls, the resulting Ct-values were taken through multi-step quality control to correct for intra-assay and inter-assay technical variability generating normalized NPX (normalized protein expression) values. Following quality control, samples that did not pass QC and deviated over 3 standard deviations from the mean IQR and or sample median in distribution plots or deviated in the principal component analysis, samples that were biological duplicates, samples that had missing serum urate data or samples that represented repeated measurements from the Treat to Target group, but had incomplete chronological timepoints (See human subjects section from STAR Methods) were excluded from further analysis (n=95) resulting in a total of 637 samples used. Proteins that had NPX values below the limit of detection in more than or equal to 20% of samples (n=19) were filtered out and excluded from further analysis, resulting in a total of 73 proteins used in the subsequent analysis. The NPX units are on a log2 scale, with 1 NPX difference equivalating to a 2-fold change in protein expression.

#### Cytokine measurements

IL-1β, IL-1Ra and IL-6 concentrations were determined using commercial ELISA kits (R&D Systems) in harvested supernatants from PBMCs treated with FGF-21 and C16+MSU or RPMI control for 24h. Absorbance was measured on Bio Tek Synergy HTX reader.

### Quantification and statistical analysis

Median and interquartile range (IQR) are used to present continuous variables. Categorical variables such as sex and presence or absence of comorbidities are presented as absolute frequencies and percent of individuals. Percentages are expressed as relative frequency of that variable based on the available data for the subgroup. Non-parametric variables age, BMI, blood creatinine, and sUA concentrations in three or more groups were tested using Kruskal-Wallis test with Dunn’s correction for multiple testing. Wilcoxon matched pairs signed rank test was used to test for differences in cytokine production between two paired groups. Categorical variables for sex and comorbidities were evaluated using a Chi-squared test. All proteomics data was corrected for age and sex using a linear regression model with age and sex as covariates. Analysis was performed using the R software and ‘stats’ package. Adjusted NPX values were retrieved and used in all downstream analyses. Untargeted group comparison analyses were performed using multiple t-test with Welch correction and two-stage step-up method of Benjamini, Krieger and Yekutieli for controlling the false-discovery rate (FDR, Q = 5%). Associations with Q <0.05 were considered significant. Targeted analysis of individual proteins for two groups comparisons was performed using Welch’s t test and Welch’s ANOVA for three groups comparison with Dunnett’s T3 test for multiple testing correction when sample size n < 50 and Games-Howell for multiple testing correction when sample size was n > 50. All tests were two-tailed, and the statistical significance cut-off was P < 0.05. All analyses were performed using GraphPad Prism (v.9.5.1) or R software^96^ (v.4.2.1).

## Consortia

The members of the HINT consortium are Leo A. B. Joosten, Ioan V. Pop, Radu A. Popp, Simona Rednic, Cristina Pamfil, Tania O. Crişan, *Marius Farcaş*, Dragoş H. Marginean, Orsolya I. Gaal, Medeea O. Badii, Ioana Hotea, Loredana Peca, Andreea-Manuela Mirea, Georgiana Cabău, Valentin Nica, Doina Colcear, Mariana S. Pop, Ancuta Rus

## Data Availability

• The Olink proteomics data have been deposited at Mendeley Data and are publicly available as of the date of publication. DOI is listed in the key resources table. • This paper does not report original code. • Any additional information required to reanalyze the data reported in this paper is available from the lead contact upon request
