# Supplementary material for: Hyperuricemia remodels the serum proteome toward a higher inflammatory state
Source: iScience. 2023 Sep 14;26(10):107909. doi: 10.1016/j.isci.2023.107909 (PMC10550725; doi:10.1016/j.isci.2023.107909)
Supplement: Document S1. Figures S1–S4 and Tables S1–S5 [file mmc1.pdf]

iScience, Volume *26*

Supplemental information

Hyperuricemia remodels the serum proteome toward a higher inflammatory state

Georgiana Cabău, Orsolya Gaal, Medeea Badii, Valentin Nica, Andreea-Manuela Mirea, Ioana Hotea, HINT-consortium, Cristina Pamfil, Radu A. Popp, Mihai G. Netea, Simona Rednic, Tania O. Cri□an, and Leo A.B. Joosten

Supplemental information

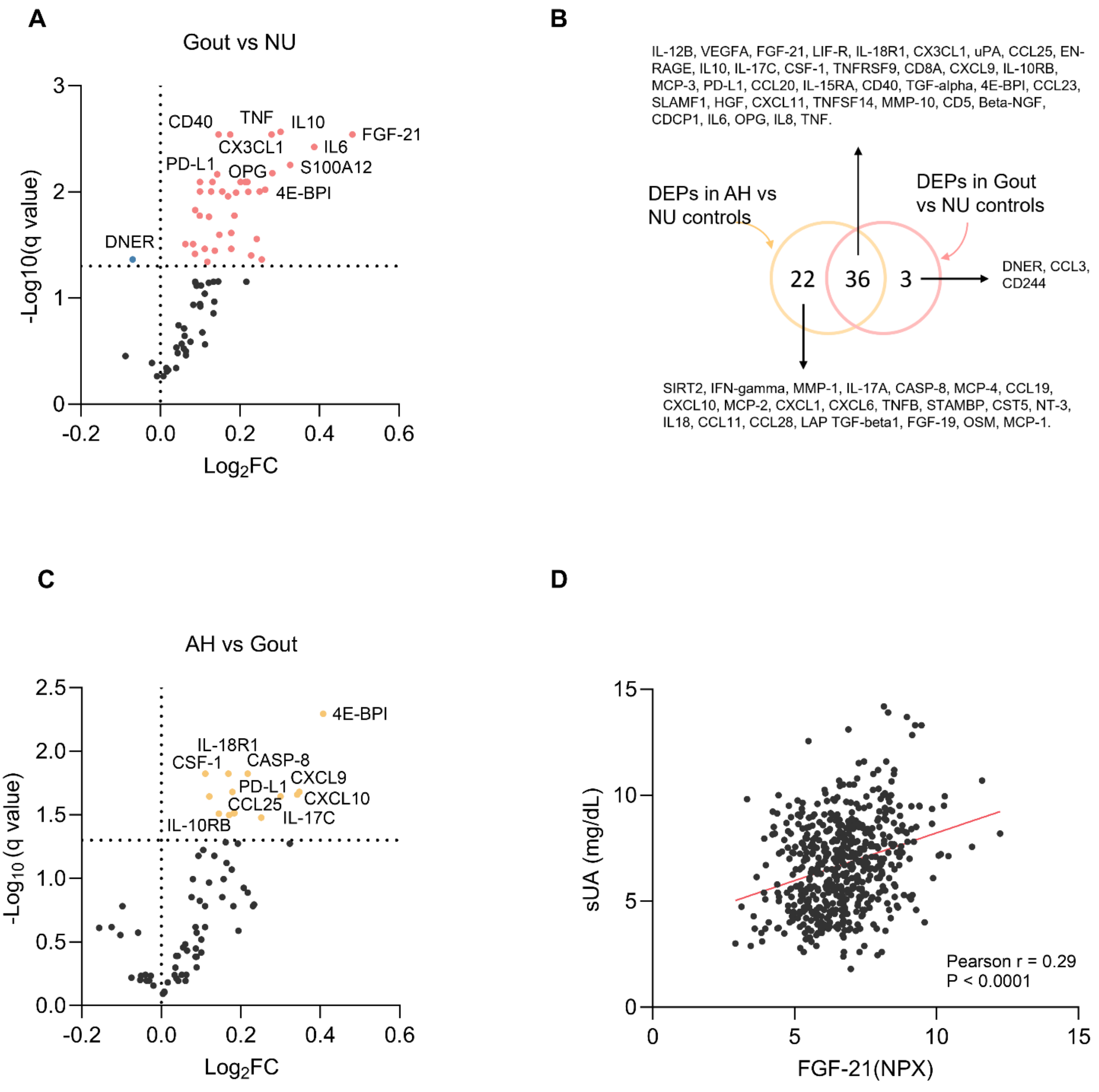

Figure S1: Serum proteomic signatures of gout and asymptomatic hyperuricemia. Related to Figure 2.

(A) Differentially expressed proteins (DEPs) in gout compared to normouricemic controls. (B) Venn diagram representing the overlap (36 proteins) between the DEPs in gout compared to NU controls (39 proteins) and asymptomatic hyperuricemia compared to NU controls (58 proteins). (C) Proteins upregulated in AH compared to gout. Volcano plots (A, C) show the top 10 proteins labeled. Associations with q values < 0.05 were considered significant, Welch multiple t-test, FDR 5%. (D) Pearson correlation between sUA levels and FGF-21 in all individuals included in the study.

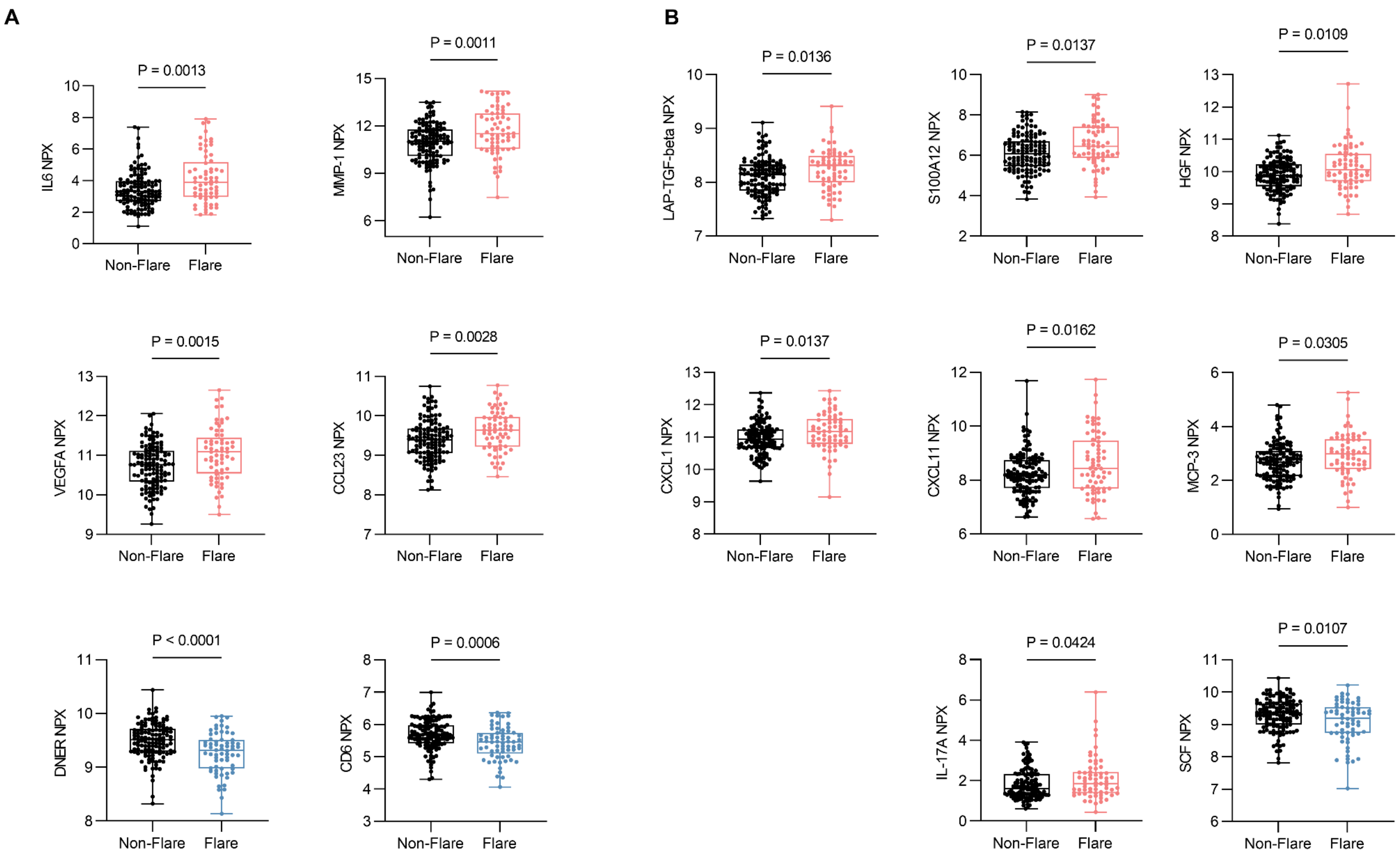

Figure S2: Differentially expressed proteins in flaring gout compared to non-flaring gout samples. Related to Figure 4.

(A) Targeted analysis of the significantly differentially expressed proteins in flaring patients. (B) Targeted analysis of the nominally significant proteins in flaring patients. Box plots show median and the 75th and 25th percentiles, whiskers show the range of values. Welch's t-test was used to compare groups. (See also Figure 4B)

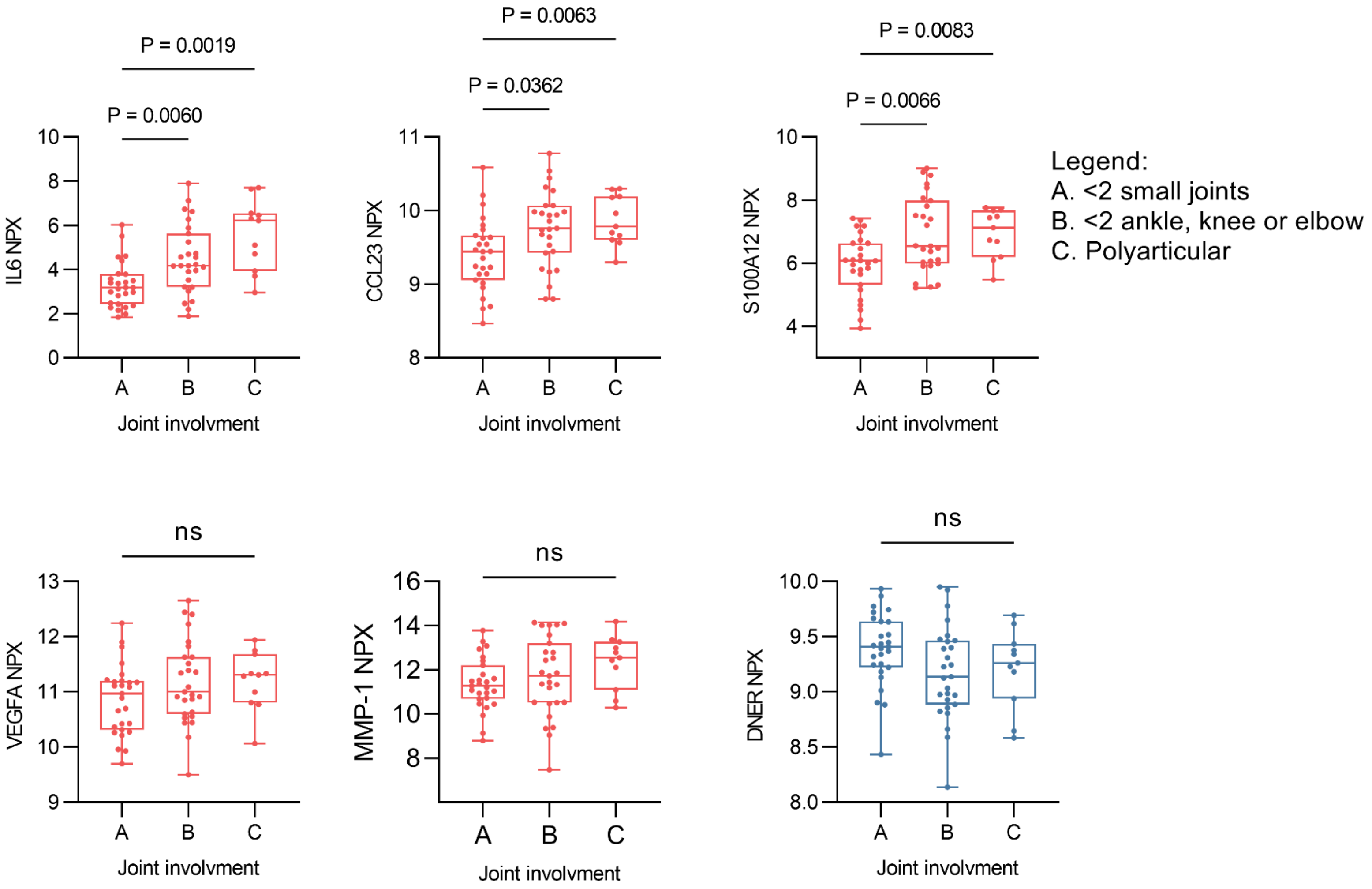

Figure S3: Protein levels of the markers associated with joint involvement in flaring gout. Related to Figure 4 and Table S3.

(A) IL-6, CCL23, S100A12 have significantly increased levels in polyarticular gout and when bigger joints were affected compared to smaller joints. Box plots show median and the 75th and 25th percentiles, whiskers show the range of values. Welch's ANOVA with Dunnett T3 for multiple testing correction was used to compare groups.

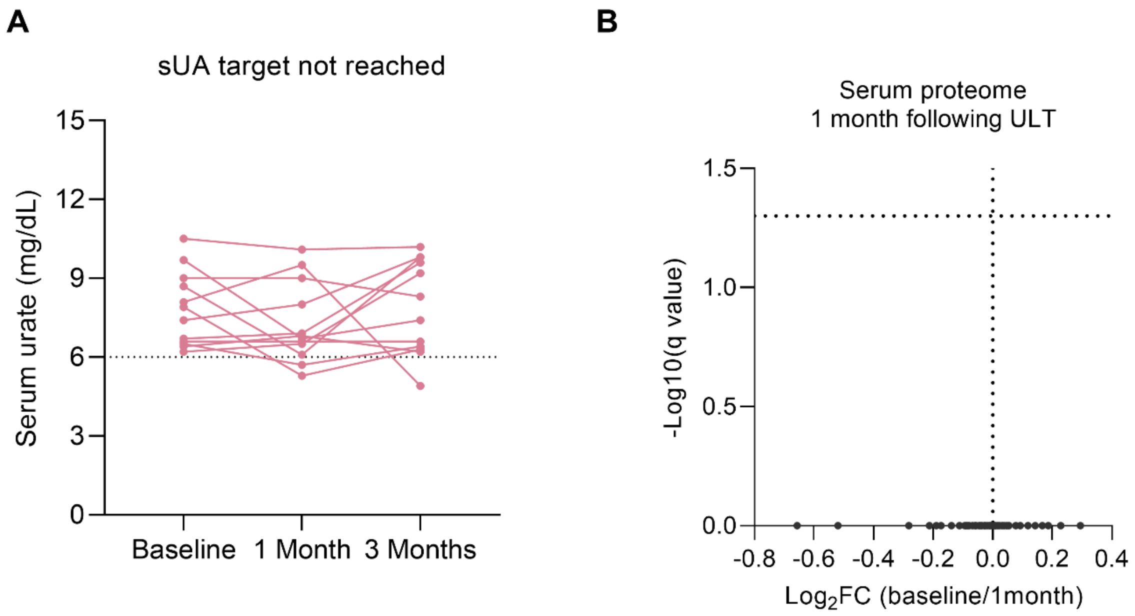

Figure S4: No early changes are observed in the proteome of patients with gout following ULT. Related to Figure 5.

(A) Patients that failed to reach sUA target (<6 mg/dL) at 1 month and 3 months compares to baseline. Dots and lines represent the paired samples at different timepoints. (b) Volcano plot showing no significant difference in proteins levels after only 1 month of ULT compared to baseline levels in patients that reached the target (sUA <6 mg/dL) after 3 months. Welch's multiple t-test, FDR 5%. (See also Figure 5).

Table S1. Prevalence of comorbidities in gout patients, hyperuricemic individuals and normouricemic controls. Related to Figure 1.

	T2DM+	T2DM-	CKD+	CKD-	CVD+	CVD-	HBP+	HBP-	Steatosis+	Steatosis-	HyperCT+	HyperCT-	HyperTG+	HyperTG-
NU n (%)	27 (12.85)	183 (87.14)	7 (3.33)	203 (96.66)	10 (4.78)	199 (95.21)	119 (56.13)	93 (43.86)	30 (14.21)	181 (85.78)	132 (62.55)	79 (37.44)	49 (23.22)	162 (76.77)
AH n (%)	48 (35.82)	86 (64.17)	35 (28.68)	87 (71.31)	23 (19.65)	94 (80.34)	112 (76.71)	34 (23.28)	53 (40.15)	79 (59.84)	79 (59.84)	53 (40.15)	64 (48.48)	68 (51.51)
Gout n (%)	30 (16.85)	148 (83.14)	25 (16.23)	129 (83.76)	14 (10.29)	122 (89.70)	135 (72.97)	50 (27.02)	65 (41.66)	91 (58.33)	89 (56.68)	68 (43.31)	82 (52.56)	74 (47.43)

NU: normouricemia; AH: asymptomatic hyperuricemia; T2DM: type 2 diabetes; CKD: chronic kidney disease; CVD: cardiovascular disease; HBP: high blood pressure; HyperCT: hypercholesterolemia; HyperTG: hypertriglyceridemia;

Table S2: Comparison between asymptomatic hyperuricemia and normouricemic controls. Related to Figure 2.

Proteins	P value	Mean of Hyperuricemia	Mean of Normouricemia	Difference	q value
4E-BPI	<0.000001	6.464	5.775	0.689	<0.000001
IL6	<0.000001	3.917	3.297	0.6205	<0.000001
IL-18R1	<0.000001	8.935	8.644	0.2916	<0.000001
CD40	<0.000001	11.81	11.52	0.2912	<0.000001
CXCL9	<0.000001	6.466	5.868	0.5976	<0.000001
PD-L1	<0.000001	6.751	6.429	0.3221	<0.000001
HGF	<0.000001	10.16	9.832	0.3262	<0.000001
CX3CL1	<0.000001	6.319	5.959	0.3602	<0.000001
IL10	<0.000001	4.893	4.383	0.5099	<0.000001
IL-17C	<0.000001	2.007	1.487	0.5208	<0.000001
CSF-1	<0.000001	10.29	10.11	0.174	<0.000001
IL-10RB	<0.000001	6.185	5.964	0.2205	<0.000001
CCL25	0.000001	6.235	5.933	0.3016	0.000002
TGF-alpha	0.000002	6.384	6.02	0.3641	0.000004
MCP-3	0.000003	2.928	2.556	0.3727	0.000005
LIF-R	0.000004	3.81	3.631	0.1794	0.000006
TNFRSF9	0.000005	6.925	6.595	0.3303	0.000007
CCL20	0.000005	6.957	6.379	0.5783	0.000007
CCL23	0.000009	9.599	9.354	0.2451	0.000011
IL-15RA	0.000012	0.9253	0.7439	0.1814	0.000014
CXCL11	0.000013	8.556	8.111	0.4452	0.000014
OSM	0.000013	7.43	7.042	0.3874	0.000014
TNF	0.00002	3.718	3.338	0.3804	0.000021
MMP-10	0.000023	6.235	5.93	0.3055	0.000023
CST5	0.000042	5.397	5.152	0.2456	0.000039
Beta-NGF	0.000042	1.499	1.34	0.1585	0.000039
CASP-8	0.00005	3.229	2.95	0.2785	0.000045
VEGFA	0.000078	10.93	10.67	0.2596	0.000067
CXCL6	0.000089	9.844	9.558	0.2861	0.000074
CXCL10	0.000117	10.32	9.873	0.4477	0.000094
CD8A	0.000129	10.26	9.964	0.3012	0.000101
FGF-21	0.000171	6.936	6.392	0.5439	0.000127
EN-RAGE	0.000174	6.341	5.926	0.4154	0.000127
OPG	0.000219	9.587	9.42	0.1668	0.000152
CDCP1	0.000221	3.25	2.958	0.2914	0.000152
CD5	0.000234	4.706	4.517	0.1884	0.000157
CXCL1	0.000433	11.14	10.92	0.2224	0.000283
LAP TGF-beta1	0.000557	8.26	8.108	0.1514	0.000354
IFN-gamma	0.001064	7.055	6.643	0.4112	0.000659
MCP-1	0.001102	12.39	12.19	0.2012	0.000665
IL18	0.00215	8.608	8.398	0.2103	0.001266
MMP-1	0.00354	11.4	11.06	0.3428	0.002035
SLAMF1	0.004062	2.939	2.757	0.1819	0.002281
IL-17A	0.005957	1.989	1.757	0.2317	0.00327
IL-12B	0.008301	6.497	6.255	0.2415	0.004455

CCL11	0.008937	7.731	7.594	0.1369	0.004692
CCL28	0.011665	2.034	1.888	0.1457	0.005994
uPA	0.017784	9.552	9.449	0.1032	0.008948
CCL19	0.022426	9.966	9.708	0.2573	0.011053
NT-3	0.031017	2.544	2.422	0.1216	0.014981
STAMBP	0.044068	4.762	4.643	0.1188	0.020868
MCP-4	0.060707	14.65	14.49	0.152	0.028194
TNFSF14	0.08277	6.375	6.234	0.1416	0.037715
IL8	0.093323	7.791	7.616	0.1749	0.041736
TNFB	0.101639	5.08	4.984	0.09634	0.044629
SIRT2	0.104679	4.502	4.349	0.1528	0.045143
FGF-19	0.110302	8.027	7.85	0.1766	0.046274
MCP-2	0.111134	10.98	10.86	0.1196	0.046274
TRAIL	0.151578	9.27	9.214	0.05667	0.062044
CD6	0.165064	5.5	5.581	-0.08134	0.066438
AXIN1	0.181652	3.049	2.951	0.09781	0.071916
ADA	0.192156	4.39	4.328	0.06237	0.074848
ST1A1	0.244796	5.421	5.583	-0.1625	0.093838
TWEAK	0.276796	10.36	10.41	-0.04733	0.104447
CD244	0.346034	6.873	6.832	0.04072	0.128088
CCL3	0.350055	7.617	7.531	0.08607	0.128088
DNER	0.355604	9.456	9.488	-0.03252	0.128177
SCF	0.382909	9.193	9.241	-0.04863	0.135989
IL7	0.472552	4.486	4.44	0.04646	0.165393
RANKL	0.520677	6.253	6.302	-0.04939	0.179634
CCL4	0.683195	7.726	7.695	0.03088	0.232383
Flt3L	0.864213	9.302	9.313	-0.01094	0.289872
CXCL5	0.881287	12.59	12.57	0.01362	0.291549

Table S3: Correlation of joint involvement with differentially expressed and nominally significant proteins in flaring gout. Related to Figure 4 and Figure S3.

Protein	Pearson r	95% C.I.	R squared	P (two-tailed)
VEGFA	0.281	0.1451 to 0.4064	0.07894	<0.0001
MCP-3	0.2257	0.08691 to 0.3560	0.05096	0.0016
TGF-beta1	0.2327	0.09423 to 0.3624	0.05416	0.0012
IL6	0.3945	0.2679 to 0.5078	0.1557	<0.0001
IL-17A	0.147	0.005476 to 0.2827	0.0216	0.0419
CXCL11	0.1931	0.05289 to 0.3258	0.03727	0.0073
CXCL1	0.2315	0.09294 to 0.3613	0.05359	0.0012
CD6	-0.2168	-0.3477 to-0.07757	0.047	0.0025
SCF	-0.2073	-0.3390 to-0.06768	0.04297	0.0039
MMP-1	0.2864	0.1509 to 0.4113	0.08201	<0.0001
HGF	0.2408	0.1027 to 0.3698	0.05797	0.0008
CCL23	0.2858	0.1503 to 0.4108	0.08169	<0.0001
DNER	-0.3234	-0.4447 to-0.1905	0.1046	<0.0001
S100A12	0.2799	0.1440 to 0.4055	0.07836	<0.0001

Table S4: Correlation of disease severity with nominally significant proteins in tophaceous gout. Related to Figure 4.

Protein	Pearson r	95% C.I.	R squared	P (two-tailed)
RANKL	0.3761	0.2477 to 0.4915	0.1414	<0.0001
MCP-3	0.214	0.07465 to 0.3451	0.0458	0.0029
TRAIL	-0.1408	-0.2769 to 0.0007920	0.01983	0.0514
IL6	0.2052	0.06554 to 0.3371	0.04212	0.0043
IL10	-0.103	-0.2411 to 0.03913	0.01062	0.1549
DNER	-0.1974	-0.3298 to-0.05743	0.03898	0.0061
CCL3	-0.1123	-0.2499 to 0.02979	0.01261	0.121

Table S5: Differentially expressed proteins between baseline and 3 months following ULT. Related to Figure 5.

Protein	P value	Mean of 3 months	Mean of Baseline	Difference	q value
LIF-R	0.000031	3.565	3.799	-0.2332	0.001788
CDCP1	0.000233	3.2	3.496	-0.2953	0.006759
IL18	0.001381	8.287	8.544	-0.2576	0.022096
NT-3	0.001524	2.399	2.714	-0.3159	0.022096
IL-10RB	0.002319	5.879	6.084	-0.2043	0.026898
CCL28	0.003164	1.994	2.285	-0.2908	0.030587
CCL11	0.004242	7.584	7.822	-0.2389	0.035152
SLAMF1	0.005926	2.852	3.089	-0.2373	0.042961
EN-RAGE	0.009862	5.682	6.51	-0.8278	0.06235
OPG	0.013472	9.506	9.727	-0.2212	0.06235
CASP-8	0.013702	2.661	3.186	-0.5257	0.06235
CD5	0.013834	4.475	4.692	-0.2177	0.06235
uPA	0.013975	9.525	9.712	-0.187	0.06235
CX3CL1	0.021435	5.976	6.18	-0.2041	0.088801
MCP-4	0.033763	14.43	14.8	-0.3739	0.130552
IL6	0.054818	3.356	3.963	-0.6062	0.198717
PD-L1	0.061516	6.441	6.622	-0.1809	0.198861
VEGFA	0.061716	10.69	10.83	-0.132	0.198861
MCP-3	0.083588	2.656	2.931	-0.2757	0.255163
IL-18R1	0.090395	8.686	8.824	-0.1378	0.262146
4E-BPI	0.097573	5.932	6.356	-0.4243	0.269488
MCP-2	0.118293	10.85	11.05	-0.207	0.309205
CSF-1	0.122616	10.13	10.22	-0.09594	0.309205
CD40	0.130427	11.51	11.63	-0.1241	0.315199
FGF-21	0.157138	6.415	6.737	-0.3228	0.364559
CXCL6	0.164819	9.577	9.778	-0.2019	0.367674
MMP-10	0.205606	5.935	6.076	-0.1404	0.422762
Beta-NGF	0.206315	1.535	1.595	-0.05947	0.422762
CCL20	0.211381	6.773	6.548	0.2246	0.422762
TNFB	0.254228	5.018	5.142	-0.1243	0.491507
IL-15RA	0.266611	0.778	0.8276	-0.04954	0.498821
CXCL10	0.296433	10.09	10.37	-0.2759	0.537284
LAP-TGF-beta1	0.31972	8.101	8.169	-0.0675	0.537913
MCP-1	0.319734	12.23	12.34	-0.1105	0.537913
CD8A	0.328796	9.826	9.938	-0.1122	0.537913
SIRT2	0.342677	4.221	4.517	-0.2965	0.537913
TNFRSF9	0.344722	6.577	6.696	-0.1184	0.537913
HGF	0.352426	9.954	10.07	-0.1137	0.537913
TGF-alpha	0.381685	6.302	6.157	0.1448	0.567635
IFN-gamma	0.442991	7.162	6.836	0.326	0.609246
IL-12B	0.449813	6.373	6.155	0.2187	0.609246
IL10	0.450437	5.071	4.738	0.3326	0.609246
CCL23	0.451682	9.374	9.44	-0.06681	0.609246
CXCL11	0.499828	8.371	8.587	-0.2158	0.658864
TNFSF14	0.534335	6.468	6.336	0.1324	0.688698

CCL25	0.563039	5.991	6.025	-0.03463	0.709919
OSM	0.577985	7.4	7.258	0.1427	0.713258
STAMBP	0.599821	4.601	4.708	-0.1064	0.724783
CST5	0.618974	5.044	5.095	-0.05063	0.726607
IL-17A	0.626385	1.651	1.573	0.07736	0.726607
TNF	0.659309	3.48	3.584	-0.1035	0.737372
CXCL9	0.661092	6.194	6.313	-0.1188	0.737372
MMP-1	0.836809	11.14	11.1	0.0412	0.894725
CCL19	0.839788	9.759	9.802	-0.04342	0.894725
FGF-19	0.863571	8.098	8.165	-0.06648	0.894725
CXCL1	0.863872	10.97	10.95	0.02096	0.894725
IL8	0.889667	7.76	7.807	-0.04625	0.905276
IL-17C	0.992079	1.505	1.508	-0.002168	0.992079
